# Web-Based Stress Management for Working Adults With Attention-Deficit/Hyperactivity Disorder (ADHD): Single-Arm, Open Pilot Trial

**DOI:** 10.2196/66388

**Published:** 2025-05-29

**Authors:** Martin Oscarsson, Sandra Hammarbäck, Karolina Blom Wiberg, Alexander Rozental, Ylva Ginsberg, Per Carlbring, Gerhard Andersson, Fredrik Jönsson

**Affiliations:** 1 Department of Psychology, Stockholm University, Albanovägen 12, Stockholm, 106 91, Sweden, 46 8 16 46 02; 2 Department of Psychiatry and Psychotherapy, University Hospital Bonn, Bonn, Germany; 3 Department of Health, Education and Technology, Luleå University of Technology, Luleå, Sweden; 4 Centre for Psychiatry Research, Department of Clinical Neuroscience, Karolinska Institutet, & Stockholm Health Care Services, Stockholm, Sweden; 5 School of Psychology, Korea University, Seoul, Republic of Korea; 6 Department of Behavioural Sciences and Learning, Linköping University, Linköping, Sweden; 7 Department of Biomedical and Clinical Sciences, Linköping University, Linköping, Sweden

**Keywords:** attention deficit disorder with hyperactivity, cognitive behavioral therapy, quality of life, pilot projects, internet-based intervention

## Abstract

**Background:**

National and international guidelines advocate for a multimodal approach to treating adult attention-deficit/hyperactivity disorder (ADHD), combining pharmacotherapy with psychological interventions. While recent reviews support cognitive behavioral therapy (CBT) as a viable treatment for ADHD in adults, evidence remains limited. Another challenge is the availability of psychological interventions, with stimulants remaining the primary treatment choice for adults with ADHD. One promising approach to increasing access to psychological interventions is the dissemination of internet-delivered CBT.

**Objective:**

This study evaluated the feasibility, acceptability, and effects of a guided web-based stress management program specifically designed for working adults with ADHD. The intervention aimed to enhance quality of life by addressing stress, exhaustion, anxiety, and depression, commonly experienced by this population.

**Methods:**

Thirty-six participants took part in a single-arm open trial, with assessments before, during, and after the intervention. The intervention consisted of 12 modules based on CBT principles, focusing on executive functioning, stress management, and emotion regulation, with clinician support on demand. Primary and secondary outcomes included quality of life (Adult ADHD Quality of Life Scale [AAQoL]), perceived stress (Perceived Stress Scale [PSS-10]), exhaustion (Karolinska Exhaustion Disorder Scale [KEDS]), anxiety (Generalized Anxiety Disorder 7-item Scale [GAD-7]), depression (Patient Health Questionnaire [PHQ-9]), and ADHD symptoms (the World Health Organization Adult ADHD Self-Report Scale [ASRS]).

**Results:**

Results indicated a statistically and clinically significant improvement in quality of life (Cohen *d*=0.84), and a reduction in ADHD symptoms (*d*=0.98), as well as statistically significant reductions in perceived stress (*d*=0.83), exhaustion (*d*=1.12), anxiety (*d*=1.70), and depression (*d*=1.25). Improvements were sustained at a 12-week follow-up. A clinically significant improvement in quality of life was observed in 36% (13/36) of participants. Participants reported high satisfaction with the program and the guidance. Adherence was high, with an overall assessment response rate of 84%, a mean of 78% of modules opened, and no explicit dropouts. Twelve of the 36 participants reported negative effects. Qualitative content analysis of participants’ written feedback revealed positive experiences and suggestions for improvement.

**Conclusions:**

This study suggests promise for web-delivered interventions tailored to the needs of adults with ADHD, pending further research and development in controlled studies.

## Introduction

Attention-deficit/hyperactivity disorder (ADHD) is a common neurodevelopmental disorder, affecting between 3% and 7% of adults worldwide [1]. Core symptoms are inattention, hyperactivity, and impulsivity. However, theoretical models propose a general deficiency in executive functions as a basis for the symptoms [2]. While the clinical presentation is well-defined in children, symptoms may have a more subtle and heterogeneous expression in adults. Despite growing evidence on the assessment and treatment of adult ADHD, many patients are underdiagnosed and undertreated, due to lack of recognition, misunderstandings, and poor access to specialized care [3,4]. Adults with ADHD have a general increased risk of experiencing mental health problems. A majority of adults with ADHD meet the criteria for at least 1 comorbid psychiatric disorder, most commonly mood, anxiety, or substance use disorders [5-7].

Adult ADHD has also been linked to stress and fatigue, and adults with ADHD generally score higher than those with no ADHD on measures of these constructs [8,9]. ADHD is also highly likely to be an underlying factor in cases of burnout [10]. In addition, adults with ADHD are more likely to experience stressful life events, such as divorce, loss of a job, and financial loss [11]. The inattentive nature of ADHD may lead to excessive proofreading, procrastination, and perfectionism, while impulsivity and lack of reflection may result in the acceptance of more tasks and responsibilities. This, combined with hyperactivity and difficulties relaxing, may lead to stress and exhaustion [12].

The impact of ADHD extends beyond individual well-being. Compared with their intellectual potential, many adults with ADHD underperform academically and professionally [13]. In most modern work environments, symptoms of inattention, hyperactivity, and impulsivity pose significant challenges [14]. In addition to impaired work performance and lower perceived effectiveness, adults with ADHD report significantly greater problems with attendance, teamwork, and social interaction at the workplace compared to controls [15]. The accumulation of performance impairment and interpersonal challenges, along with symptoms of psychiatric comorbidities, may significantly diminish the quality of life of adults with ADHD [16,17]. Additional negative outcomes include productivity losses and costs associated with sickness absence [18]. Several studies, such as the one by de Graaf et al [19], have indicated that adults with ADHD have more sickness absence days than controls, with some reporting rates twice [20] or several times as high [21].

National and international guidelines, such as those by Kooij et al [3] and the National Institute for Health and Care Excellence [22], advise a multimodal approach for the treatment of adult ADHD, combing pharmacotherapy with psychological interventions. Psychological interventions for adult ADHD include psychoeducation and cognitive behavioral therapy (CBT). While recent reviews, such as those by Fullen et al [23] and Nimmo-Smith et al [24], lend support to CBT as a viable treatment of ADHD in adults, evidence is limited due to a lack of available research and significant methodological issues with the included studies. Another limitation of CBT and other psychological interventions for adult ADHD is that of availability, with stimulants, in practice, remaining the treatment of choice for adults with ADHD [3].

While pharmacological treatment may be effective in reducing core symptoms of ADHD, functional impairment often remains. When discussing CBT for adults with ADHD and potential mechanisms of change, Ramsay [25] considered Barkley’s theory of executive functioning and self-regulation [26,27]. Executive functions, such as behavioral inhibition, verbal and nonverbal working memory, and emotional self-regulation, are necessary for a person when organizing and executing goal-directed actions. Impaired executive functioning in adult ADHD interferes not only with the actual management of everyday tasks and activities, but also with the ability to modify dysfunctional behaviors associated with the disorder. In short, most adults with ADHD know what needs to change in terms of their behavior but fall short in implementing and maintaining changes. Consequently, Ramsay [25] characterized ADHD as an implementation disorder.

One way to increase access to psychological interventions is the dissemination of internet-delivered cognitive behavioral therapy (iCBT) [28]. iCBT with minimal clinician support has been shown to yield similar effects to face-to-face therapy for several psychiatric and somatic disorders, although repeated direct comparisons remain limited to a few specific conditions [29]. In a recent review and meta-analysis by Svärdman et al [30], iCBT was shown effective in reducing self-reported stress, as well as anxiety and depressive symptoms, among adults experiencing stress and stress-related disorders. iCBT has also been evaluated for adults with ADHD. In a recent randomized controlled trial (RCT) by Nasri et al [31], guided iCBT was found to yield significantly greater improvement on the main ADHD outcome measure than a treatment-as-usual control. In another recent RCT, Kenter et al [32] found that an unguided web intervention (MyADHD) based on CBT, dialectical behavior therapy and goal management training, was superior to a psychoeducation-only control in a sample of adults with ADHD. Earlier RCTs on adult ADHD include 1 by Pettersson et al [33], showing that unguided iCBT was superior to a waiting list control, and 1 by Moëll et al [34], showing that a CBT-inspired guided web-based course was superior to a waiting list control.

In a recent qualitative study on stress- and work-related psychological problems among adults with ADHD [35], participants reported few and unsatisfactory experiences of health care support beyond pharmacological treatment of the ADHD symptoms. Several participants specifically mentioned a desire for interventions designed for patients with a relatively high level of functioning, with an emphasis on executive functions. While psychoeducational interventions for adult ADHD may include information and exercises related to workplace functioning, such as in the study by Hirvikoski et al [36], it is not a primary focus in most common ADHD interventions. In fact, a recent review by Lauder et al [37] failed to identify any ADHD interventions specifically designed with the workplace context and workplace functioning as a central component.

The limited offering and inadequate provision of psychological interventions for working adults with ADHD are particularly conspicuous when considering the high prevalence of ADHD and the potential of resource-efficient iCBT. Inspired by existing psychological interventions for ADHD, stress, and common psychiatric comorbidities, and informed by interviews with working adults with ADHD, we developed a guided web-based stress management program specifically for the adult population with ADHD. The program was based on CBT principles, aiming to enhance quality of life by addressing stress, exhaustion, anxiety, and depression. In this pilot study, we evaluate the feasibility, acceptability, and effects of this novel intervention.

## Methods

### Design

This was a single-arm open trial with repeated measures before, during, and after the intervention. The primary aim was to evaluate the feasibility, acceptability, and effects of a guided web-based stress management program for working adults with ADHD.

### Preregistration

The research question, desired sample size, included variables, and planned main analysis were preregistered on Open Science Framework [38]. Preregistration was completed during the recruitment phase before any participants had started the intervention.

### Participants

Information about the study was shared on social media by a large Swedish ADHD patient interest group (Riksförbundet Attention), who posted about the study on their Facebook page, and by the research team, who posted in relevant Facebook groups and on LinkedIn. The study was conducted entirely on the web, without involvement of any physical or clinical sites. All recruitment, screening, informed consent, and data collection took place via the secure web intervention platform iTerapi [39]. Accompanying the recruitment information was a link to a site on the iTerapi platform, where those interested were invited to read more about the study. The site provided details including participation criteria and a button to create an account and submit a notice of interest in participation. Provision of informed consent was required to submit a notice of interest.

Upon the creation of an account, prospective participants were sent a link to validate their email address and to complete a web-based screening questionnaire. The screening questionnaire included questions regarding sociodemographics and ADHD diagnosis, as well as self-report measures of quality of life, perceived stress, symptoms of exhaustion, anxiety, depression, and symptoms of ADHD. The questionnaire also included questions regarding possible concurrent psychological or pharmacological treatment of ADHD or mental health problems.

To be eligible for the study, participants needed to be aged 20‐64 years (Swedish definition of working age), fluent in Swedish, have a diagnosis of ADHD, and work half-time or more. They also needed to score >19 on the Perceived Stress Scale (PSS-10; 75th percentile [40]), or >18 on the Karolinska Exhaustion Disorder Scale (KEDS; at risk for exhaustion disorder [41]), or >4 on the Generalized Anxiety Disorder 7-item Scale (GAD-7; mild anxiety [42]), or >9 on the Patient Health Questionnaire (PHQ-9; moderate depression [43]). Participants were excluded in cases of severe depression (PHQ-9>19), suicidality (PHQ-9 item 9>1), concurrent psychological treatment, start of psychological treatment scheduled for the next 3 months, psychotropic medication dosage not stable for at least 1 month, or change in psychotropic medication scheduled for the next 3 months.

In total, 47 individuals created an account on the treatment platform. One did not complete the screening questionnaire, while 4 met the exclusion criteria (1 on sick leave, 1 with a recent change in psychotropic medication, 1 with a scheduled change in psychotropic medication, and 1 with a score above 19 on the PHQ-9). The remaining 42 were invited to participate in the study, and 36 accepted the invitation. Table 1 shows the characteristics of the final sample.

**Table 1.** Demographic and clinical characteristics of participants (n=36) in a single-arm pilot trial of a web-based stress-management intervention for working adults with attention-deficit/hyperactivity disorder in Sweden.

Characteristics	Participants (n=36)
Age (years), mean (SD)	44.4 (8.29)
Gender, n (%)	
Women	28 (78)
Men	7 (19)
Other or did not want to assign	1 (3)
Education level, n (%)	
Elementary school	0 (0)
High school	4 (11)
University ≤3 years	7 (19)
University >3 years	25 (69)
Employment rate (percentage of full-time), mean (SD)	92.9 (11.7)
Psychotropic medication, n (%)	
Yes	31 (86)
No	5 (14)

### Measures

The main outcome measure was quality of life, measured with the Adult ADHD Quality of Life Scale (AAQoL [44]). Secondary outcome measures were perceived stress, measured with the PSS-10; symptoms of exhaustion, measured with the KEDS; anxiety, measured with the GAD-7; depression, measured with the PHQ-9; and symptoms of ADHD, measured with the World Health Organization Adult ADHD Self-Report Scale (ASRS) Symptoms Checklist [45]. The frequency and time points of measurement are shown in Table 2. Week 12 is postintervention and week 24 is follow-up. Subscale results are reported for the PSS-10 and the ASRS. The ASRS was modified to reflect the last 4 weeks, and formatted without the shaded boxes, as is common in ADHD intervention research. The Negative Effects Questionnaire (NEQ-20 [46]) was used to explore negative effects and adverse events.

**Table 2.** Overview of outcome measures and assessment time points during a 12-week guided web-based stress-management intervention and 12-week follow-up for working adults with attention-deficit/hyperactivity disorder in Sweden.

Measure	Baseline	Week 2	Week 4	Week 6	Week 8	Week 10	Week 12	Week 24
AAQoL^a^	√	N/A^b^	√	N/A	√	N/A	√	√
ASRS^c^	√	N/A	√	N/A	√	N/A	√	√
PSS-10^d^	√	N/A	√	N/A	√	N/A	√	√
KEDS^e^	√	√	√	√	√	√	√	√
PHQ-9^f^	√	√	√	√	√	√	√	√
GAD-7^g^	√	√	√	√	√	√	√	√
NEQ-20^h^	N/A	N/A	N/A	N/A	N/A	N/A	√	N/A

a AAQoL: Adult Attention-Deficit/Hyperactivity Disorder Quality of Life.

b N/A: not applicable.

c ASRS: Adult ADHD Self-Report Scale.

d PSS-10: Perceived Stress Scale.

e KEDS: Karolinska Exhaustion Disorder Scale.

f PHQ-9: Patient Health Questionnaire.

g GAD-7: Generalized Anxiety Disorder 7-item Scale.

h NEQ-20: Negative Effects Questionnaire.

At week 12, participants were also invited to answer open-ended questions about their experiences of the intervention and rate their treatment satisfaction from 1 to 4 on 7 items.

### Intervention

The intervention, called “Working with ADHD,” was developed to increase the quality of life for working adults with ADHD by addressing and managing stress, exhaustion, anxiety, and depression. The program is based on CBT principles, incorporating psychoeducation, basic behavior analysis, goal setting, and simple behavioral experiments. The development process began with a comprehensive review of existing psychological interventions targeting ADHD, stress, and common psychiatric comorbidities.

As part of a previous qualitative study [35], 20 working adults with ADHD were asked about their interest in and attitudes toward a hypothetical web-based psychological intervention developed specifically for working adults with ADHD. In addition to being generally positive toward the concept, the participants underscored 3 things that would increase the likelihood of its success: treatment content presented in short, accessible modules with clear rationale; the option to choose modules based on needs and urgency; and the inclusion of clinician support.

The intervention had 3 main areas of focus. The first was compensatory strategies for executive function deficiencies, with a foundation in Barkley’s theory of ADHD and Ramsay’s proposed mechanisms of change. This included information and exercises regarding organization, planning, and prioritization, inspired by the CBT manual for adult ADHD by Safren et al [47]. Also included were information and exercises from CBT for procrastination, inspired by interventions developed by Rozental et al [48].

The second area of focus was stress management and recovery. Here, the theoretical basis was the cognitive behavioral model of clinical burnout by Almén [49]. Information and exercises revolved around the balance between stress reactivity and stress recovery, with an emphasis on recovery-facilitating behaviors. This also included information and exercises regarding sleep, inspired by the ADHD-specific behavioral insomnia intervention by Jernelöv et al [50].

The third and final area of focus was emotion regulation. The role of emotional dysregulation in the clinical presentation of adult ADHD has been extensively discussed in the literature, illustrated, for example, by the study by Hirsch et al [51]. Emotional dysregulation, as a component of adult ADHD, has also been linked to occupational impairment [52]. It may take different forms, including a hypersensitivity to criticism and failure [35], as well as impatience and irritability [52]. In this intervention, information and exercises revolved around assertiveness and self-esteem [53], as well as perfectionism [54] and self-compassion [55].

The intervention content was delivered in 12 modules over 12 weeks on the iTerapi platform. The platform is responsive and usable on computers, tablets, and smartphones. First, participants were granted access to a new module every Monday for 4 weeks. Then, participants were granted access to 6 new modules, to work on for 6 weeks, according to their preference and perceived needs. Finally, participants were granted access to 2 new modules, weeks 11 and 12, respectively. This is detailed in Table 3.

**Table 3.** Overview of the 12-module web-based stress management intervention (“Working with ADHD”) delivered over 12 weeks to working adults with attention-deficit/hyperactivity disorder in Sweden.

Module	Theme	Content
1	ADHD^a^, stress, self-assessment, and goals	ADHD, stress, and mental health; assessment of current situation; and goal formulation
2	Communication and boundary setting	Performance-based self-esteem, assessment of and working with self-esteem, self-compassion, regulating strong emotions, and communicating boundaries
3	Sleep	ADHD and sleep, sleep assessment, sleep hygiene, and sleep restriction
4	Recovery	The recovery paradox, assessment, recovery every day, practicing self-awareness, and finding balance in everyday life
5	Planning and prioritization	Use of calendar and to-do list, and prioritizing tasks
6	Problem-solving	Stepwise problem-solving, breaking tasks into smaller parts, and attention span
7	Perfectionism	Perfectionism, stress, and ADHD; assessment of perfectionist behaviors; practicing flexible thinking; and seeing the positive
8	Procrastination	Procrastination, stress, and ADHD; assessment; motivation; minimum effort; and managing distractions
9	Disclosing the diagnosis	Job seeking with ADHD and disclosing the diagnosis at the workplace
10	Rights and responsibilities at the workplace	General rights and responsibilities of employees; and the right to accommodation
11	Repetition and reflection	Summary of previous modules, assessment of what has been helpful, and noting progress
12	Beyond the program	Setbacks and relapses; and identifying risk situations

a ADHD: attention-deficit/hyperactivity disorder.

All modules were structured into brief sections, each alternating between information, assessment, and exercises. Most modules included 1500‐2000 words in total. Participants were instructed to complete all assessments and exercises in writing, using their preferred medium (eg, notes app or spiral notebook). At the conclusion of each module, participants were offered suggestions for implementing new strategies in their daily lives. In addition, select sections within each module included prompts for participants to reach out to the research group for support.

Support was available on demand through asynchronous messaging on the treatment platform. All support was provided by a licensed psychologist with 5 years of clinical experience working with adults with ADHD. The support primarily consisted of encouragement and affirmation [56] and did not introduce new treatment content. Each participant also received 2 scheduled, prewritten messages per week: one on Mondays containing information about new modules or offering general encouragement to engage with the treatment content and another later in the week encouraging participants to reach out with questions or request feedback on the exercises. Messages from participants were answered at least once every weekday, that is, within 36 hours.

All participants received email notifications for new messages. If a participant had not logged in for a week, they were first contacted via email the following Monday and then twice by telephone later in the week. Participants were contacted via email and telephone in a similar manner if they did not respond to assessments. Study staff were immediately notified if a participant at any point scored PHQ-9 >19 (severe depression cutoff) or PHQ-9 item 9 >1 (suicidality cutoff), in which case the participant was contacted via telephone.

### Analysis

Linear mixed models were fitted to estimate the fixed effects of time and the random effects of participant intercepts and participant slopes, using all available data from screening to postintervention. The models used unstructured covariance patterns and restricted maximum likelihood estimation for missing data. Paired-samples *t* tests (2-tailed) were used to compare full-scale follow-up scores with screening scores using last observation carried forward (LOCF) imputation. Effect sizes Cohen *d* were calculated on estimated random means for screening to postintervention, and raw means for screening to follow-up, using the mean differences and the pooled SDs.

The Jacobson and Truax [57] method was used to analyze clinical significance, using cutoff A (ie, 2 SDs in the direction of a functioning distribution) and reliable change index (RCI) on screening and postintervention AAQoL and full-scale ASRS data with LOCF imputation. Deterioration was defined as exceeding the RCI in a negative direction, while unchanged corresponded to a change score not exceeding the RCI. Analysis of missing data was done using one-way ANOVA. Adherence, treatment satisfaction, negative effects, and adverse events were explored descriptively. All statistical analyses were performed using jamovi (version 2.4; The jamovi project) [58,59]. Mixed models were run using the GAMLj module [60]. Jacobson-Truax classifications and RCI were calculated using the JTRCI R package [61].

Inductive qualitative content analysis [62] was used to explore data from participants’ responses to the open-ended questions postintervention. The analysis was conducted by SH and KBW, under the supervision of MO. SH and KBW were final-year psychologist students at the time of the analysis. They had completed basic training in CBT and previously assisted MO in developing the treatment content. The analysis followed the procedure outlined by Krippendorff [63]. First, data were sampled from the participants, using the open-ended questions. Data were unitized, using the original wording of the participants and discarding data not relevant to the research questions. Then, the units of meaning were coded inductively, using the analysts’ own understanding of the context. Finally, the codes were clustered, maintaining internal homogeneity and external heterogeneity between the final categories.

### Ethical Considerations

This study was approved by the Swedish ethical review authority (diary nos.: 2022-04370-01 and 2022-07214-02), and all procedures followed relevant national and institutional guidelines. Informed consent was obtained digitally from all participants via the secure iTerapi platform, prior to the submission of interest in participation. Participants were provided with written information about the study, data handling, and their rights, and were encouraged to contact the principal investigator (FJ) with any questions before consenting. Study data were handled in pseudonymized form using a coded key, accessible only to the research team. All personal data were stored securely on encrypted servers or encrypted researcher devices (eg, university computers), either within the treatment platform or Stockholm University’s approved storage systems. No financial compensation was provided to participants. No identifiable images or personal information is shown in the manuscript or Multimedia Appendix 1.

## Results

### Main Analyses

**Figure 1.**
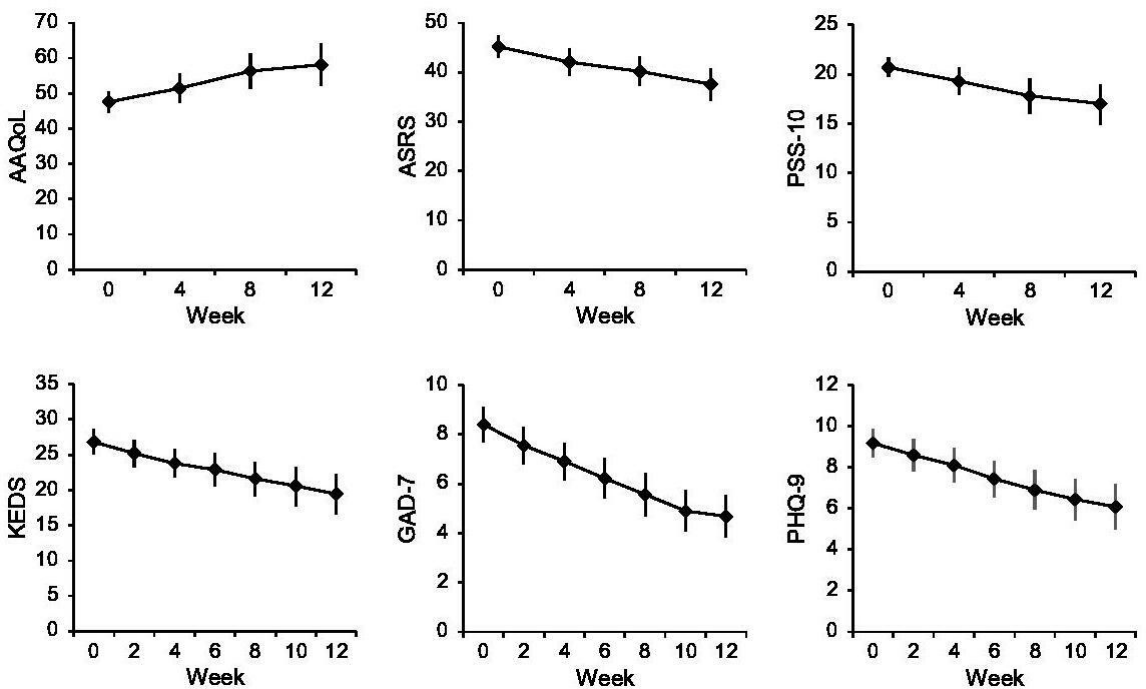
Estimated marginal means with 95% CIs for all outcome measures from screening to postintervention in a single-arm pilot trial of a guided web-based stress-management intervention for working adults with attention-deficit/hyperactivity disorder in Sweden. AAQoL: Adult Attention-Deficit/Hyperactivity Disorder Quality of Life; ASRS: Adult Attention-Deficit/Hyperactivity Disorder Self-Report Scale; GAD-7: Generalized Anxiety Disorder 7-item Scale; KEDS: Karolinska Exhaustion Disorder Scale; PHQ-9: Patient Health Questionnaire-9; PSS-10: Perceived Stress Scale-10.

A mixed model revealed a statistically significant effect of time from screening to postintervention on the main outcome measure AAQoL (*F*_1,29.4_=28.2; *P*<.001), corresponding to a large effect (*d*=0.84). This indicates an improved average quality of life among the participants. Mixed models also revealed statistically significant effects of time from screening to postintervention on the secondary outcome measures, all indicating lower average symptom levels among participants. The results of the mixed models, with corresponding effect sizes Cohen *d*, are shown in Table 4. Estimated marginal means are shown in Figure 1 and Table S1 in Multimedia Appendix 1.

**Table 4.** Results from linear mixed models estimating changes in all outcome measures from screening to postintervention in a single-arm pilot trial of a guided web-based stress management intervention for working adults with attention-deficit/hyperactivity disorder in Sweden.

Measure	*F* test (*df*)			*P* value	Cohen *d*
AAQoL^a^	28.2 (1, 29.4)			<.001	0.84
ASRS^b^	35.6 (1, 29.8)			<.001	0.98
ASRS (Inattention)	30.9 (1, 30.8)			<.001	1.12
ASRS (H/I)	28.8 (1, 28.8)			<.001	0.72
PSS-10^c^	17.3 (1, 31.5)			<.001	0.83
PSS-10 (Negative)	28.6 (1, 31.0)			<.001	1.01
KEDS^d^	26.1 (1, 33.5)			<.001	1.12
PHQ-9^e^	22.4 (1, 33.0)			<.001	1.25
GAD-7^f^	53.1 (1, 28.7)			<.001	1.70

a AAQoL: Adult Attention-Deficit/Hyperactivity Disorder Quality of Life.

b ASRS: Adult Attention-Deficit/Hyperactivity Disorder Self-Report Scale.

c PSS-10: Perceived Stress Scale-10.

d KEDS: Karolinska Exhaustion Disorder Scale.

e PHQ-9: Patient Health Questionnaire-9.

f GAD-7: Generalized Anxiety Disorder 7-item Scale.

Paired-samples *t* tests indicate statistically significant differences between week 24 follow-up scores and screening scores on all outcome measures, corresponding to large effect sizes Cohen *d*. This indicates an improved average quality of life and lower average symptom levels among participants, 12 weeks postintervention. The results of the paired-samples *t* tests, with corresponding effect sizes Cohen *d*, are shown in Table 5. LOCF means are shown in Table S2 in Multimedia Appendix 1.

**Table 5.** Paired-samples *t* test results comparing outcome measures between screening and follow-up in a single-arm pilot trial of a guided web-based stress management intervention for working adults with attention-deficit/hyperactivity disorder in Sweden.

Measure	*t* test (*df*)		*P* value	Cohen *d*
AAQoL^a^	4.32 (35)		<.001	0.83
ASRS^b^	−5.36 (35)		<.001	0.82
PSS-10^c^	−5.89 (35)		<.001	1.12
KEDS^d^	−5.46 (35)		<.001	0.99
PHQ-9^e^	−5.36 (35)		<.001	1.31
GAD-7^f^	−5.32 (35)		<.001	1.13

a AAQoL: Adult Attention-Deficit/Hyperactivity Disorder Quality of Life.

b ASRS: Adult Attention-Deficit/Hyperactivity Disorder Self-Report Scale.

c PSS-10: Perceived Stress Scale-10.

d KEDS: Karolinska Exhaustion Disorder Scale.

e PHQ-9: Patient Health Questionnaire-9.

f GAD-7: Generalized Anxiety Disorder 7-item Scale.

### Clinical Significance

On the primary outcome measure AAQoL, 6 of the 36 participants were recovered, 1 nonreliably recovered, 7 improved, 20 unchanged, and 2 deteriorated. On the ASRS, 3 participants were recovered, 1 nonreliably recovered, 10 improved, and 22 unchanged. Individual participant outcomes and Jacobson-Truax classifications are shown in Figures S1 and S2 in Multimedia Appendix 1.

### Adherence

There were no explicit dropouts, that is, no participants communicated a premature discontinuation of treatment. However, adherence varied between participants in the case of login frequency, number of modules opened, and number of measurements completed. The mean number of logins was 17.7 (SD 9.28) and the total assessment response rate was 84%. The mean number of modules opened was 9.31 (SD 3.86). All participants but 1 opened the 2 introductory modules, and 29 of the 36 participants opened at least half of the modules. The mean number of messages sent by participants to study staff was 3.4 (SD 2.5).

### Analysis of Missing Data

One-way ANOVA showed no statistically significant difference between screening scores of participants who did or did not respond to the measurement at week 12. This indicates that there were no apparent baseline differences between responders and nonresponders.

### Negative Effects

Twenty-nine of the 36 participants completed the NEQ-20 at week 12, and 33% (12/36) of the participants reported at least 1 negative effect likely to have been caused by the intervention. The most common negative effects, caused by the intervention or not, were the feeling of more stress (n=10) and the feeling that the treatment did not produce any results (n=5).

### Treatment Satisfaction

Twenty-nine of the 36 participants completed the treatment satisfaction questionnaire at week 12. The results are shown in Table 6.

**Table 6.** Self-rated treatment satisfaction at postintervention (week 12) among participants in a guided web-based stress management intervention for working adults with attention-deficit/hyperactivity disorder in Sweden.^a^

Items	Values, mean (SD)
1. How do you assess the quality of the treatment you have received?	2.90 (0.67)
2. To what extent have the modules been relevant to you and your areas of concern?	3.03 (0.63)
3. How satisfied are you with the opportunity to ask questions about the content of the modules?	3.48 (0.63)
4. How satisfied are you with the length of the treatment?	3.14 (0.74)
5. Has the treatment helped you to better cope with your problems?	3.00 (0.60)
6. Overall, how satisfied are you with the treatment you have received?	3.03 (0.63)
7. If a friend needed similar help, would you recommend our treatment to him or her?	3.17 (0.60)

a 1=dissatisfied, 2=indifferent, 3=mostly satisfied, and 4=very satisfied.

### Qualitative Results

Twenty-seven of the 36 participants answered the open-ended questions about their experiences of the intervention. Qualitative content analysis yielded 2 main categories and 7 subcategories that describe the participants’ experiences. These are shown in Table 7.

**Table 7.** Main categories and subcategories from qualitative content analysis of participants’ written feedback at postintervention in a pilot trial of a guided web-based stress management intervention for adults with attention-deficit/hyperactivity disorder in Sweden.

Categories and subcategories	Content
Helpful parts of the intervention	
Communication	Good, inviting, effective, appreciated reminders. “Very good. Generous and inviting, with personal feedback provided when necessary in the follow-up surveys” (Participant G).
Module content	Good, relevant, concise, easy to understand, some particularly appreciated content. “That I could see connections between performance-based self-esteem and stress. Reminded of the importance of recovery, which I apparently had completely skipped” (Participant B).
Treatment platform	Good, easy to navigate, modules (continuous publication, optional modules), login (secure). “Great setup regarding how the modules were released and the opportunity to choose what felt relevant” (Participant C).
Desired development of the program	
More structure	Checklists, guidance, templates, check-ins, clearer guidance, one thing at a time, control questions, too little structure toward the end, emphasize that tasks are not followed up, difficult without a deadline. “Having treatment over the internet was both good and bad for me. It would have been helpful if what I did was somehow checked off. One of my difficulties is getting things done without a deadline. At the same time, perhaps I wouldn’t have had the opportunity to complete all tasks if there were deadlines” (Participant C).
More audiovisual	Layout, more user-friendly, visual materials, audio for tired eyes, being able to listen, images. “In a way, it’s nice that the graphics are incredibly stripped down and boring, although maybe not so inspiring. Could certainly have benefited from a little more love” (Participant I).
Clinician support	Video calls, personal contact, short digital check-ins, clinician support for structure. “A brief conversation that provides drive to continue” (Participant J).
Technical functionality	Easier navigation, writing directly on the platform, filling in directly on the platform, digital workbook, more interactive. “More templates and mandatory check-ins. Checklists are very helpful” (Participant K).

## Discussion

The purpose of this study was to evaluate the feasibility, acceptability, and effects of a web-based stress management program developed specifically for adults with ADHD. The results showed statistically significant improvements on all outcome measures, from preintervention screening to postintervention and follow-up assessments. The change in mean self-reported quality of life from screening to postintervention corresponds to a large effect, while changes in mean scores on secondary outcome measures correspond to medium-to-large effects. Participants expressed satisfaction with the program content, platform, and communication. More structure, support, audiovisual content, and technical improvements were requested.

The statistically significant increase in self-reported quality of life, corresponding to a large effect, indicates that the participants experienced increased quality of life over the course of the intervention. As previously noted, the cumulative impact of performance impairment, interpersonal challenges, and psychiatric comorbidities significantly diminishes the quality of life of adults with ADHD. The current intervention targets common psychiatric comorbidities among adults with ADHD, with a specific focus on workplace functioning that could affect work performance. Thus, the observed increase in quality of life among the participants, over the course of the intervention, may signify a reversal of this cumulative burden, as supported by concurrent improvements on measures of perceived stress and psychiatric symptoms, corresponding to medium-to-large effects. These findings imply that the intervention’s content and delivery are well suited and beneficial for working adults with ADHD experiencing elevated levels of perceived stress, exhaustion, anxiety, and depression.

The observed pre-post effect sizes on both the AAQoL and the secondary outcome measures of psychiatric symptoms were notably larger than within-group effect sizes reported in previous studies. In the MyADHD studies by Nordby et al [64] and Kenter et al [32], improvements on the AAQoL corresponded to medium effects (*d*=0.62 and *d*=0.60, respectively), compared with *d*=0.84 in this study. Here, it should be noted that the MyADHD intervention was unguided, without clinician support. This could indicate the potential added value of clinician involvement in web-delivered interventions, enhancing their efficacy beyond what is achievable in unguided formats [65]. On PSS measures, Nordby et al [64] reported *d*=0.35 (PSS-14), Kenter et al [32] reported *d*=0.20 (PSS-14), and Moëll et al [34] reported *d*=0.03 (PSS-10), while the means observed by Nasri et al [31] correspond to an effect size of *d*=0.37 (PSS-4), compared with *d*=0.83 (PSS-10) in this study. Similarly, on the GAD-7 and the PHQ-9, Nordby et al [64] reported *d*=0.04 and *d*=0.32, respectively, compared with *d*=1.70 and *d*=1.25 in this study.

Although ADHD symptoms were not a primary target of the current intervention, the ASRS means indicate a statistically significant improvement pre-post, corresponding to large effects on the total scale (*d*=0.98) and the inattention subscale (*d*=1.12) and a medium effect on the hyperactivity subscale (*d*=0.72). These effect sizes are more in-line with within-group effect sizes reported in previous studies. Nordby et al [64] and Kenter et al [32] reported total ASRS effect sizes of *d*=0.93 and *d*=0.80, respectively, while the means reported by Nasri et al [31] correspond to a smaller effect size of *d*=0.68. Moëll et al [34] reported similarly large effects on the inattention (*d*=1.18) and hyperactivity (*d*=0.55) subscales. Again, MyADHD was unguided, and both MyADHD and the web-based course by Moëll et al [34] focused primarily on symptoms of inattention. Considering that clinician support may have played a role in the superior outcomes observed in this study compared with unguided interventions, future research could directly compare the efficacy of interventions with and without support, or investigate the potential of generative artificial intelligence acting as a clinician. This could determine whether such technology could enhance intervention effectiveness through personalized support and real-time engagement, particularly for adults with ADHD [66].

Participants were invited to rate their satisfaction and provide feedback through open-ended questions regarding their experiences with the intervention. Overall, satisfaction scores were positive, particularly regarding the opportunity to inquire about treatment content. Responses to the open-ended questions highlighted participants’ positive perceptions of the communication with the research group, describing it as good, inviting, and effective. Ramsay [25] underscores the therapist’s role in CBT for adult ADHD, validating frustrations and aiding patients in reengaging with the change process. The incorporation of support on demand in the current intervention aimed to mitigate premature discontinuation of treatment due to frustration among the participants. Encouragingly, none of the participants explicitly withdrew from the study, although adherence varied. However, the mean number of modules opened (78%) exceeds the reported number of completed modules for the unguided intervention of Nordby et al (62%) [64] and Kenter et al (65%) [32], as well as for the guided interventions of Moëll et al (53%) [34], Pettersson et al (61%) [33], and Nasri et al (56%) [31]. The average number of messages sent by the participants to study staff was 3.4 messages. This is in-line [67] or higher [68] than in previous iCBT trials with support on demand. However, postintervention, several participants suggested improved and increased support in future development of the program.

When discussing the results of this study, it is essential to acknowledge the absence of any control group. This means the design did not control for the expectation of benefit, other common factors of digital psychological interventions, or the natural disease progression [69]. Also important to note are the characteristics of the sample, which primarily consisted of highly educated women, with a large majority on psychotropic medication. This may limit the generalizability of the findings to other adult populations with ADHD, particularly men and nonbinary individuals, those with lower levels of education, and those not on psychotropic medication. Predominately female samples are common in iCBT trials, as exemplified by the study by Lindner et al [70]. In Sweden, women also show a small majority among adults with ADHD [71]. However, future trials would benefit from exploring other recruitment strategies, including ones targeting specific populations, to achieve more representative samples. Future trials would also benefit from higher-resolution measures of pharmacological treatment among participants, for example, separating ADHD prescriptions from other psychotropic medication.

Considering the study’s strengths, inclusion criteria were generous and did not exclude individuals with autism, learning disabilities, or psychiatric comorbidities other than severe depression or suicidality. While this makes for a sample more representative of the heterogeneous adult ADHD population, the demographic range is still limited. The outcome measures are well established and psychometrically evaluated. In light of recent criticism of the PSS [40], subscale outcomes were reported in the results. The potential issues with the KEDS, recently shown by Lindsäter et al [41], should be mentioned. However, the scale remains the benchmark for web intervention research in Sweden, and it is supplemented by the other measures included in the study. Concerning the measures, inclusion criteria required only an above-cutoff score on one of the secondary outcome measures. This means that not all participants had elevated levels of stress, exhaustion, anxiety, and depression at screening. This could introduce floor effects, as some participants lacked room for improvement on 1 or more measures. This is also why RCI was calculated and presented for only the AAQoL and the ASRS, relevant for all participants.

The intervention was based on previous interventions that have been shown efficacious for ADHD symptoms and common ADHD comorbidities. The intervention content was designed with ADHD theory and mechanisms of change in mind. The development of the intervention was also preceded by interviews with adults with ADHD, regarding workplace functioning and the intervention proposition. From the results of this study, it is clear that the intervention has the potential to be helpful for adults with ADHD who are experiencing stress, exhaustion, anxiety, and depression. Based on this, and the feedback provided by the participants, the intervention will be revised before being further evaluated in an RCT. If proven helpful and effective, the intervention could be developed further and possibly disseminated to a broader population through routine health care or as a stand-alone product, such as an app, as exemplified in the studies by Selaskowski et al [72] and Seery et al [73].

## Supplementary material

10.2196/66388 Multimedia Appendix 1 Estimated marginal means, follow-up means, and Jacobson-Truax plots.

## Abbreviations

AAQoL: Adult Attention-Deficit/Hyperactivity Disorder Quality of Life Scale
ADHD: attention-deficit/hyperactivity disorder
ASRS: Adult Attention-Deficit/Hyperactivity Disorder Self-Report Scale
CBT: cognitive behavioral therapy
GAD-7: Generalized Anxiety Disorder 7-item Scale
iCBT: internet-delivered cognitive behavioral therapy
KEDS: Karolinska Exhaustion Disorder Scale
LOCF: last observation carried forward
NEQ: Negative Effects Questionnaire
PHQ-9: Patient Health Questionnaire
PSS: Perceived Stress Scale
RCI: reliable change index
RCT: randomized controlled trial
