# Supplementary material for: Web-Based Stress Management for Working Adults With Attention-Deficit/Hyperactivity Disorder (ADHD): Single-Arm, Open Pilot Trial
Source: JMIR Form Res. 2025 May 29;9:e66388. doi: 10.2196/66388 (PMC12140506; doi:10.2196/66388)
Supplement: Multimedia Appendix 1 [file formative-v9-e66388-s001.pdf]

Table S1. Estimated marginal means (and standard deviations) for outcome measures across seven time points (weeks 0–12) in a single-arm pilot trial of a guided web-based stress-management intervention for working adults with attention-deficit/hyperactivity disorder in Sweden.

Measure	Week 0		Week 2		Week 4		Week 6		Week 8		Week 10		Week 12	
	M	*SD*	*M*	*SD*	*M*	*SD*	*M*	*SD*	*M*	*SD*	*M*	*SD*	*M*	*SD*
AAQoL	47.6	8.93	-	-	51.5	11.1	-	-	56.3	13.0	-	-	58.2	15.4
ASRS	45.2	6.80	-	-	42.1	7.71	-	-	40.2	7.71	-	-	37.6	8.66
ASRS (Inattention)	24.6	3.12	-	-	23.0	3.47	-	-	21.7	3.82	-	-	20.6	3.96
ASRS (H/I)	20.7	4.79	-	-	19.1	5.42	-	-	18.5	5.11	-	-	16.9	5.73
PSS-10	20.7	3.03	-	-	19.3	3.73	-	-	17.8	4.65	-	-	17.0	5.49
PSS-10 (Negative)	14.3	2.35	-	-	13.1	2.82	-	-	11.8	3.40	-	-	11.1	3.82
KEDS	26.8	5.18	25.2	5.32	23.8	5.52	22.9	6.04	21.6	6.41	20.6	7.06	19.4	7.61
PHQ-9	9.17	1.99	8.59	2.13	8.10	2.24	7.44	2.31	6.89	2.45	6.43	2.61	6.07	2.90
GAD-7	8.40	2.14	7.55	2.08	6.91	2.08	6.21	2.16	5.56	2.20	4.90	2.18	4.68	2.24

Table S2. Means and standard deviations at baseline (week 0) and follow-up (week 24), using last observation carried forward (LOCF) imputation, in a single-arm pilot trial of a guided web-based stress-management intervention for working adults with attention-deficit/hyperactivity disorder in Sweden.

Measure	Week 0		Week 24	
	M	*SD*	*M*	*SD*
AAQoL	47.6	10.8	56.2	12.6
ASRS	46.5	8.58	39.0	9.62
PSS-10	20.8	4.47	15.2	5.41
KEDS	28.1	6.55	20.7	8.23
PHQ-9	9.97	3.46	5.53	3.31
GAD-7	9.56	4.30	5.31	3.11

Figure S1. Individual participant scores on the Adult ADHD Quality of Life Scale (AAQoL) at baseline and postintervention, with Jacobson-Truax classifications applied, in a single-arm pilot trial of a guided web-based stress-management intervention for working adults with attention-deficit/hyperactivity disorder in Sweden.

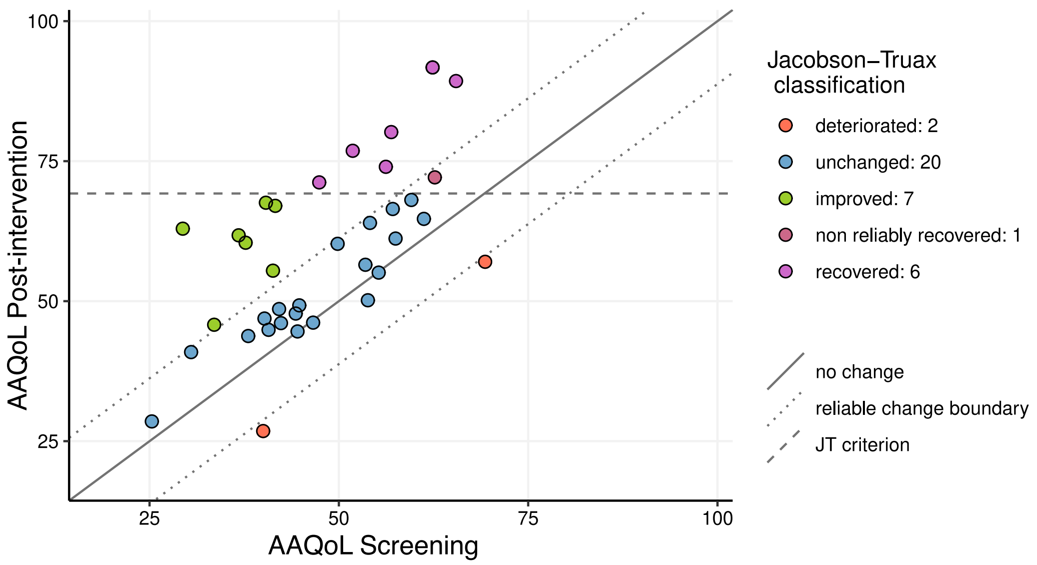

Note. Jacobson-Truax Criterion A=69.2 (two standard deviations from the baseline sample mean).

Figure S2. Individual participant scores on the World Health Organization Adult ADHD Self-Report Scale (ASRS) at baseline and postintervention, with Jacobson-Truax classifications applied, in a single-arm pilot trial of a guided web-based stress-management intervention for working adults with attention-deficit/hyperactivity disorder in Sweden.

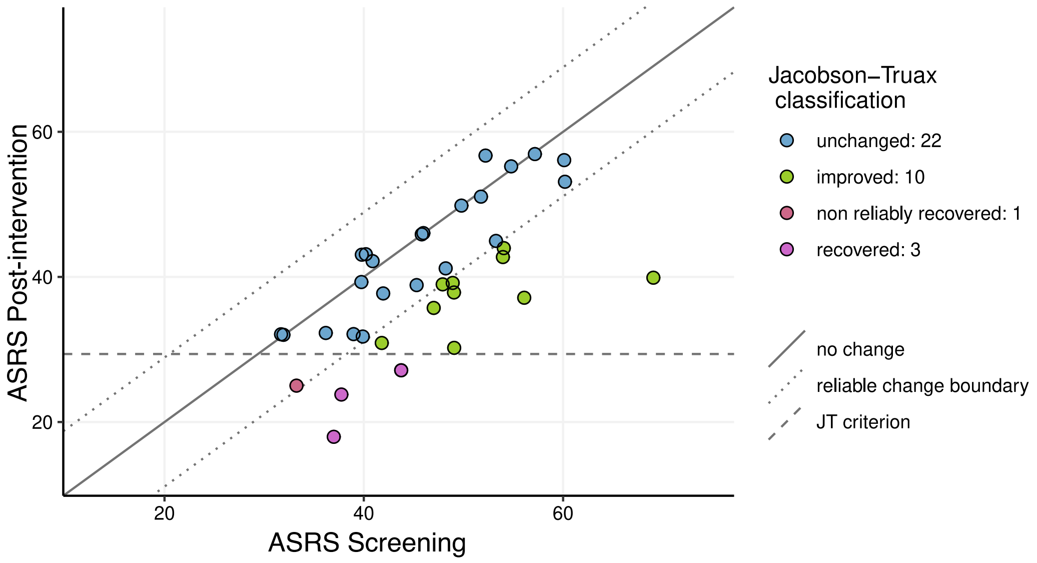

Note. Jacobson-Truax Criterion A=29.4 (two standard deviations from the baseline sample mean).
